# Expression profiling of single cells and patient cohorts identifies multiple immunosuppressive pathways and an altered NK cell phenotype in glioblastoma

**DOI:** 10.1111/cei.13403

**Published:** 2019-12-16

**Authors:** H. J. Close, L. F. Stead, J. Nsengimana, K. A. Reilly, A. Droop, H. Wurdak, R. K. Mathew, R. Corns, J. Newton‐Bishop, A. A. Melcher, S. C. Short, G. P. Cook, E. B. Wilson

**Affiliations:** ^1^ Leeds Institute of Medical Research at St James's, University of Leeds School of Medicine, St James's University Hospital Leeds UK; ^2^ MRC Medical Bioinformatics Centre University of Leeds Leeds UK; ^3^ Department of Neurosurgery Leeds General Infirmary Leeds UK; ^4^ Institute of Cancer Research London UK

**Keywords:** glioblastoma, immune‐inhibition, NK cells

## Abstract

Glioblastoma (GBM) is an aggressive cancer with a very poor prognosis. Generally viewed as weakly immunogenic, GBM responds poorly to current immunotherapies. To understand this problem more clearly we used a combination of natural killer (NK) cell functional assays together with gene and protein expression profiling to define the NK cell response to GBM and explore immunosuppression in the GBM microenvironment. In addition, we used transcriptome data from patient cohorts to classify GBM according to immunological profiles. We show that glioma stem‐like cells, a source of post‐treatment tumour recurrence, express multiple immunomodulatory cell surface molecules and are targeted in preference to normal neural progenitor cells by natural killer (NK) cells *ex vivo*. In contrast, GBM‐infiltrating NK cells express reduced levels of activation receptors within the tumour microenvironment, with hallmarks of transforming growth factor (TGF)‐β‐mediated inhibition. This NK cell inhibition is accompanied by expression of multiple immune checkpoint molecules on T cells. Single‐cell transcriptomics demonstrated that both tumour and haematopoietic‐derived cells in GBM express multiple, diverse mediators of immune evasion. Despite this, immunome analysis across a patient cohort identifies a spectrum of immunological activity in GBM, with active immunity marked by co‐expression of immune effector molecules and feedback inhibitory mechanisms. Our data show that GBM is recognized by the immune system but that anti‐tumour immunity is restrained by multiple immunosuppressive pathways, some of which operate in the healthy brain. The presence of immune activity in a subset of patients suggests that these patients will more probably benefit from combination immunotherapies directed against multiple immunosuppressive pathways.

## Graphical abstract

Our manuscript challenges the current paradigm that Glioblastoma (GBM) is a poor candidate for immunotherapy, therefore impacting on clinical approaches. We identify NK cells as a target for immunosuppression in GBM, with multiple suppressive pathways within GBM human tumours. We also identify a group of GBM patients with higher immunological activity, who will benefit from immunotherapies that target these immunosuppressive pathways.

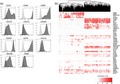

## Introduction

Glioblastoma (GBM) is the most common and aggressive type of primary adult brain cancer. Current treatments include debulking neurosurgery and adjuvant chemo/radiotherapy. Despite these therapies, median overall survival is just 12–24 months 1. Recent developments in cancer immunotherapy provide one potential approach to improve patient outcomes 2. However, despite significant therapeutic impact on several solid tumour types, immune checkpoint blockade (ICB) is yet to demonstrate benefit in GBM treatment 3.

Evasion of host immunity is a hallmark of cancer 4. Tumours exploit the negative feedback mechanisms that the healthy immune system uses to dampen immune responses. These mechanisms include the recruitment of immune cells with suppressive activity, the expression of immunosuppressive cytokines, such as transforming growth factor (TGF)‐β and immune checkpoints, including programmed cell death (PD)‐1 and cytotoxic T lymphocyte antigen (CTLA)‐4 5. Chronic interactions between tumour cells and infiltrating T cells leads to an exhausted phenotype, an unresponsive but reversible state with an altered transcriptional profile 6. Exhausted tumour‐infiltrating lymphocytes express immune checkpoints, and antibodies that target these molecules can reinvigorate anti‐tumour immunity 7.

Mutations in the tumour genome are a source of neoantigens and mutation frequency is a surrogate marker for immunogenicity 8. For several tumours, neoantigen load is correlated with survival and with response to immune checkpoint blockade; melanoma and lung adenocarcinoma have higher mutational load, greater T cell infiltration, greater PD‐1 expression and consequently show better responses to anti‐PD‐1 therapy 9. Compared to other solid tumours, mutation frequency and T cell infiltration levels in GBM are low. However, GBM‐infiltrating T cells have been isolated against a number of germline‐encoded antigens over‐expressed in the tumour, indicating that T cell responses are at least possible 10. Glioblastoma cells are a target for natural killer (NK) cells; however, the number of GBM‐infiltrating natural killer (NK) cells is also low 11. The paucity of NK cells and T cells in GBM is compounded by the high proportion of suppressive myeloid‐lineage cells 12 able to suppress lymphocyte function 5.

Importantly, GBM‐infiltrating T cells express PD‐1 13, yet reports of initial anti‐PD‐1 (nivolumab) clinical trials are not encouraging 2. This indicates that PD‐1 expression *per se* is not sufficient to allow responsiveness to therapy, and that additional suppressive components of the GBM immune landscape regulate many effectors of anti‐tumour immunity.

Here we show that, *in vitro*, GBM cells are recognized and killed by NK cells; however, NK cells derived from GBM tumours have an altered cell surface phenotype consistent with their inhibition in the tumour microenvironment. We have explored the basis of this inhibition and identify numerous immunosuppressive mechanisms operating in GBM contributed by both tumour and immune cell compartments. These immunosuppressive pathways, some of which appear to operate in the normal brain, are a barrier to effective immunotherapy, but also represent candidate therapeutic targets to reinvigorate tumour NK cell interactions.

## Materials and methods

### Ethics statement

Ethical approval for this study was granted by the ethics committee at the Leeds Teaching Hospitals NHS Trust, Leeds UK (REC number 10‐H1306‐7).

### Classification of GBM patients consensus immune cluster (CIC)

CIC classification 14 was applied to GBM tumour transcriptome data from The Cancer Genome Atlas (TCGA). Briefly, consensus cluster analysis of melanomas used the expression of 380 genes specific to 24 immune cell types (15; this produced six subtypes which we termed CICs. The average expression of each gene within each CIC is the cluster centroid. Using TCGA data, we used the nearest centroid method 16 to classify each GBM tumour into one of the CICs according to the highest Spearman’s correlation coefficient with the centroids. For each of the 24 immune cell types of the immunome compendium 15, we calculated a score per GBM tumour, graphically represented using a heatmap.

### Differential expression of genes in GBM CIC2 and CIC4 and in REMBRANDT data

To compare the expression of selected genes in different patient groups we used RNAseq data from TCGA [obtained via The Cancer Imaging Archive (TCIA) 7], assigning patients to either CIC2 or CIC4 (as above). In addition, we used microarray data from the REMBRANDT study 17 downloaded from Betastasis.com. For REMBRANDT, patient samples were classified as granzyme A (GZMA)^high^ expressors or GZMA^low^ expressors based on the median expression value (*n* = 214). Expression of selected genes was compared between CIC2 and CIC4 (for TCGA data) or the GZMA^high^ and GZMA^low^ patients (for REMBRANDT) and analysed using non‐parametric, unpaired statistical testing (using GraphPad Prism).

### Single‐cell data and normal brain analysis

Single‐cell (sc)RNAseq data were downloaded from 18 and expression of candidate genes analysed. Data were visualized using rStudio version 1.0.143 (package: gplots 3.0.1) using heatmap.2. Untransformed data clustering (unsupervised) was performed (Euclidean distance). Individual cells were classified as ‘tumour’ or ‘immune’ according to co‐expression of SRY‐box transcription factor 9 (SOX9) and epidermal growth factor receptor (EGFR) 18 and protein tyrosine phosphatase receptor type C (PTPRC), respectively. For all genes, expression of > 0 was scored positive. For immune (PTPRC^+^) and tumour (SOX9^+^EGFR^+^) cells, the number of different immunomodulatory molecules expressed was counted for each cell and the percentage of immune and non‐immune cells expressing immunomodulatory genes plotted. Expression of individual genes in non‐tumour‐bearing brain tissue was downloaded from 19. These data are also available (with graphical output) at BrainRNAseq.org.

### Tumour tissue and blood, collection and processing

After ethical approval and informed consent, tumours were resected and stored in phosphate‐buffered saline (PBS) or within the cavitron ultrasonic surgical aspirator (CUSA) 20. Samples were washed in PBS, CUSA samples were prepared as shown by Schroeteler *et al.* 20 and all samples were filtered through a 40‐µm cell strainer, washed twice in PBS, centrifuged at 400 ***g*** for 5 min and resuspended in PBS, 0·5% bovine serum albumin (BSA) and 0·05% sodium azide. Matched patient blood was diluted with PBS, layered over Ficoll (Axis‐Shield PoC, Oslo, Norway) and centrifuged at 800 ***g*** for 20 min. Tumour and blood‐derived cells were stained with appropriate antibodies and isotype controls (see Supporting information, Table S1), with single stain controls on tumour samples used for compensation during analysis using the cytexpert compensation matrix. All samples were run on a CytoFlex S (Beckman Coulter Life Sciences, Indianapolis, IN, USA) (see Supporting information, Table S1). Gated, isotype control stained, intratumoral or peripheral blood NK cells from each patient (Supporting information, Fig. S1) were assigned a gate of 2% positive, and specific antibody staining is reported within this gate.

### Primary cells and cell lines

Neural progenitor cells (NP1) were isolated from a patient undergoing surgery to treat epilepsy 21. The primary lines, GBM1 and NP1, were generated at the Scripps Institute. GBM11, GBM13 and GBM20 were derived at the University of Leeds using the same method and culture conditions 22. Peripheral blood mononuclear cells (PBMC) were isolated from whole blood of healthy donors as above. NK cells were further separated using an NK cell isolation kit (Miltenyi Biotec, Bergisch Gladbach, Germany), and cultured in Dulbecco’s modified Eagle’s medium (DMEM) supplemented with 10% fetal bovine serum (FBS) and 10% human AB serum (Sigma‐Aldrich, Gillingham, UK).

### Surface antigen screening

GBM stem‐like cell (GSC) lines were harvested using 0·25% trypsin/ethylenediamine tetraacetic acid (EDTA) and fluorescently labelled for 60 min at 37°C and 5% CO_2_ in serum‐free media with one of the the following cell dyes: 0·4 μM cell tracker^TM^ (CT)‐green CMFDA (488 nm excitation), 2 μM CTorange‐CMRA (488 nm excitation), 2 μM CTviolet‐BMQC (407 nm excitation) or 5 μM calcein blue‐AM (407 nm excitation) (all from Invitrogen, Carlsbad, CA, USA) All populations were washed three times, mixed together and plated at a density of 1 × 10^6^ total cells/well in 96‐well round‐bottomed plates (Nunc, Roskilde, Denmark). Cells were stained as per the manufacturer’s instructions with 242 antibodies from the BD Bioscience Lyoplate screening panel, followed by Zombie NIR (Biolegend, San Diego, CA, USA) for 30 min before resuspension and analysis by flow cytometry. Cells were gated based on their emitting fluorescence at 520 nm (CTgreen loaded), 580 nm (CTorange loaded), 540 nm (CTviolet loaded) or 449 nm (calcien blue loaded). The median fluorescence intensity (MFI) for each gated population, for each antigen and isotype control emission at 668 nm (Alexa647 emission) was generated and GSC lines scored as positive if more than 20% of the population expressed the antigen. Flow cytometer and settings are as described earlier; analysis was performed using FacsDiva (BD Biosciences, San Jose, CA, USA), FlowJo (Treestar, Inc., Ashland, OR, USA) and Kaluza (Beckman Coulter) software.

### Natural killer cytotoxicity assays

Target tumour cell lines were labelled with the relevant cell dye (see surface screen) for 1 h at 37°C, washed twice and plated at 2 × 10^5^/well. NK cells were pre‐activated with 20 ng/ml interleukin (IL)‐15 for 48 h and mixed with targets at the E : T ratios indicated. After 5 h, cells were pelleted (300 ***g*** for 5 min), washed with PBS and stained with Zombie NIR (Biolegend) for 15 min at room temperature. Competitive cytotoxicity assays were set up as above; the two target cell types under test (GBM and neural progenitors) were labelled with either CTgreen or CTviolet, mixed 1 : 1 and used as a target population at an E : T of 5 : 1.

## Results

### Glioma stem‐like cells are effective targets of NK cells

Effective therapy for GBM will require the elimination of the radioresistant GSCs that are largely responsible for recurrence 23. While tumour‐associated antigen‐specific T cells offer a highly selective therapeutic approach, antigen‐independent effector cells, such as NK cells, have the potential to target and destroy GBM tumour cells that have a low neoantigen load.

**Figure 1:**
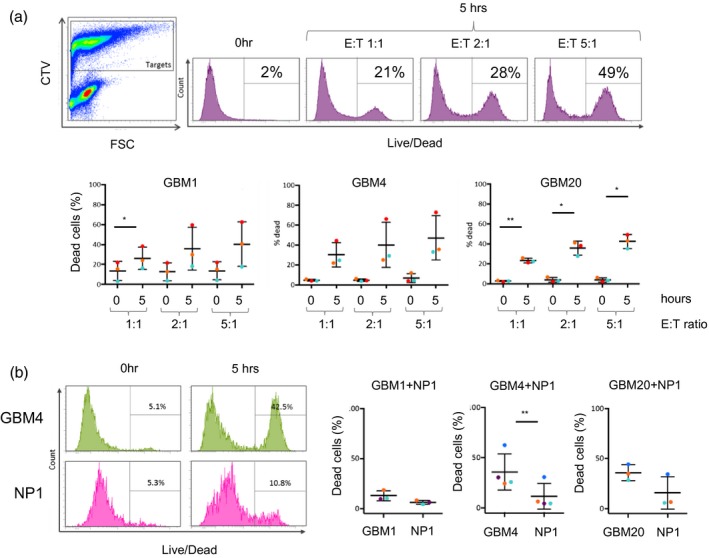
Natural killer (NK) cell‐mediated killing of glioma stem‐like cells. (a) NK cell cytotoxicity: cell tracker violet‐labelled glioblastoma (GB) stem‐like cell (GSC) lines (targets) were co‐cultured with unlabelled, interleukin (IL)‐15 activated NK cells (effectors) for 5 h at effector : target (E : T) ratios as shown. Co‐cultures were then stained with a live/dead discriminator. The panel on the left shows identification of effector and target cells in the co‐culture (for gating purposes) and the panels to the right show death of the labelled target cells at the different E : T ratios. The zero hour control is included as background cell death of the GSC cells. The three graphs summarize data obtained using three GSC lines (GBM1, GBM4 and GBM20) and three different NK cell donors (coloured dots), with standard deviation from the mean. (b) NK cell specificity: cytotoxicity assays of IL‐15‐activated NK cells co‐cultured with a 1 : 1 mix of the GSC line (indicated) and neural progenitor cells (NP). The GSC and NP lines were labelled with different cell tracker dyes, allowing their fate in the assay to be determined separately. The flow cytometry plots show the percentage of dead GSC (here GBM4) and NP cells after zero and 5 h co‐culture with NK cells. The graphs summarize these data for assays containing the three GSC lines using NK cells from four separate donors (coloured dots), with standard deviation from the mean.

We used three patient‐derived GSC lines 24 shown to exhibit a stem cell‐like expression profile and recapitulate high‐grade gliomas in orthotopic xenograft mouse models 22, 24 and performed cytotoxicity assays using peripheral blood‐derived, IL‐15‐activated NK cells to confirm NK cell‐mediated killing. Tumour cells differentiated from GSCs are more sensitive to NK cell killing than the GSC themselves 26, but GSCs are killed by NK cells in the presence of activating cytokines (Fig. 1a) 25. We further tested whether NK cells activated with IL‐15 would be efficient killers of GSCs, but retain specificity for GSCs over normal neural progenitor cells. We performed an NK cytotoxicity assay using a mixed target cell population comprised of tumour GSC cells and normal neural progenitor (NP) cells 22 at a ratio of 1 : 1. For all donors, IL‐15‐activated NK cells killed tumour cells in preference to the NP cells (Fig. 1b). These results suggest that, in short‐term *in‐vitro* cultures when sufficient immune cells are present and activated, GSCs are an effective and preferential target for NK cells.

### Patient‐derived NK cells exhibit an altered cell surface phenotype in GBM

**Figure 2:**
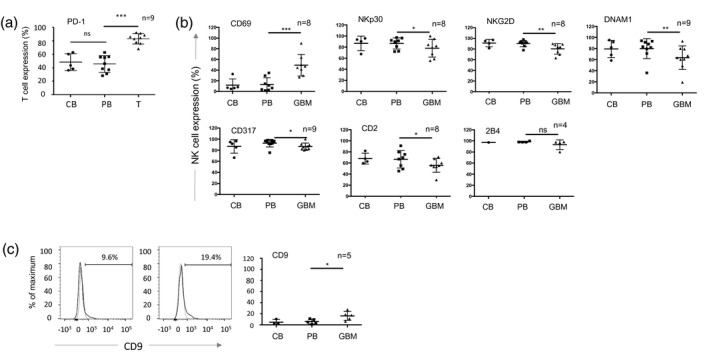
The cell surface phenotype of glioblastoma (GBM)‐infiltrating lymphocytes. (a) Expression of programmed cell death 1 (PD‐1) on CD3^+^ T cells in GBM patient tumour (GBM), patient blood (PB) and control blood from healthy donors (CB). Each dot represents a single patient sample (*n* is the number of GBM patient samples analysed); the bar indicates the mean ± standard deviation. The patient‐derived tumour (GBM) and blood (PB) samples were analysed using a paired *t*‐test; *P < 0·05, **P < 0·01; ***P < 0·001; n.s. = not significant. (b) Expression of NK cell surface molecules (gating on CD45^+^, NKp46^+^, CD3^neg^ cells) in GBM patient tumour, patient blood and control blood from healthy donors as in (a). (c) Representative histograms of CD9 expression on PB and GBM‐derived natural killer (NK) cells, grouped data as in (a). Statisical analysis was performed using a paired *t*‐test. *P < 0·05, **P < 0·01.

The presence of infiltrating NK cells in GBM 11, coupled with their ability to recognize and kill GSCs (Fig. 1), suggests that they are rendered non‐functional in the GBM tumour microenvironment. We performed flow cytometry‐based analysis of intratumoural NK from GBM tissue and compared their surface phenotype to NK cells derived from autologous peripheral blood as well as blood from healthy donors. NK cell populations were defined as NKp46^+^ and CD3^–^ due to high expression of CD56 (NCAM‐1) on GBM tumour cells within the sample (Supporting information, Fig. S1). To confirm sampling of immune cells from within the GBM tumour tissue (and not from blood contamination of the tumour sample) we assayed the expression of PD‐1 on T cells, and showed significantly enhanced expression of PD1 on tumour‐derived T cells compared to their blood counterparts (Fig. 2a). Expression levels of NK cell surface molecules were similar on the blood‐derived NK cells from both healthy donors and GBM patients. However, the expression of the tumour‐sensing NK cell‐activating receptors NKp30, NKG2D and DNAX accessory molecule‐1 (DNAM‐1) and the surface molecules tetherin/CD317 and CD2 were all significantly reduced on the GBM tumour‐derived NK cells compared to those from matched peripheral blood (Fig. 2b). Together with higher expression of PD‐1 on GBM‐derived T cells compared to matched peripheral blood (Fig. 2a), we also found higher expression of lymphocyte‐activation gene (LAG‐3) and CTLA‐4 (although differences in CTLA‐4 expression did not reach statistical significance) (Supporting information, Fig. S2).

Members of the TGF‐β family are highly expressed in GBM, and are important in maintaining the GSC pool 25. Furthermore, we and others have previously shown that TGF‐β reduces the expression of NKp30, NKG2D and DNAM‐1 on NK cells and is associated with their functional inactivation 26, 27. Importantly, TGF‐β induces the expression of the tetraspanin CD9 on NK cells 28, and we detected significantly increased expression of CD9 on the surface of the GBM‐resident NK cells compared to NK cells from matched peripheral blood (Fig. 2c). The reduced expression of NK cell activating receptors coupled with the increased expression of CD9 is suggestive of TGF‐β‐mediated evasion of NK cell cytotoxicity in the GBM microenvironment.

Collectively, GBM resident immune effector cells clearly demonstrate two separate phenotypes: the reduced expression of NK cell activation receptors and the increased expression of immune checkpoint molecules on T cells.

### Surface antigen screening of GSCs identifies candidate immunomodulatory molecules

**Figure 3:**
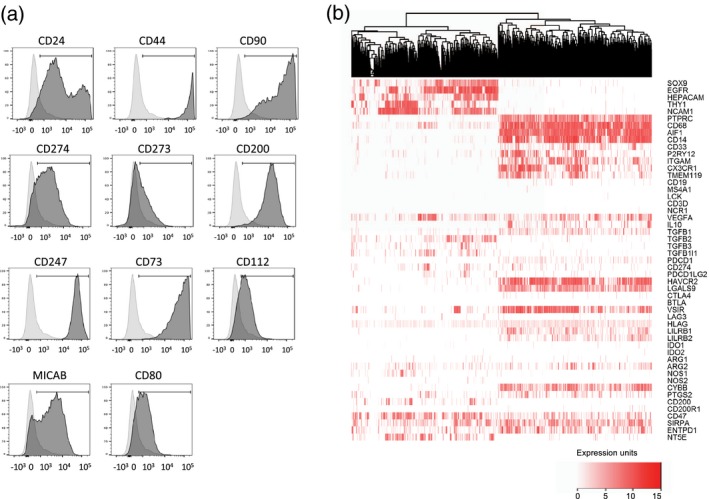
The repertoire of immunosuppressive molecules expressed in glioblastoma (GBM). (a) Expression of selected cell surface antigens on GBM stem‐like cell (GSC) lines; the data show expression by GBM20. A summary of expression across the four GSC lines is provided in Supporting information, Table S1. (b) Single‐cell (sc) RNAseq data [18] were clustered, revealing immune and tumour groups marked by protein tyrosine phosphatase receptor type C (PTPRC) and SRY‐box transcription factor 9 (SOX9/EGFR co‐expression, respectively. Expression of marker genes for cell lineages and those encoding immunomodulatory molecules are indicated. Expression is scored according to the values and key shown.

The GSC lines are selectively targeted by NK cells *in vitro*, but evade NK cells and other immune effector cells *in vivo*. To understand which immunomodulatory molecules expressed by GSC might be responsible for immune activation and inhibition, we analysed GSCs for the expression of cell surface immunomodulatory molecules. Using a flow cytometry‐based screen, we identified 116 cell surface antigens expressed on four GSC lines lines (Supporting information, Table S1). Molecules detected on the GSCs included those associated with the cancer stem cell phenotype (CD24, CD44 and CD90) (Fig. 3a), as well as widely expressed cell surface molecules, such as major histocompatibility complex (MHC) class I (and β2‐microglobulin), CD71 and CD98, as expected. Several immune inhibitory molecules were highly expressed, such as the immune checkpoint ligands programmed cell death ligand 1 (PD‐L1) (CD274) and PD‐L2 (CD273), providing a source for inhibition of PD‐1 expressing T cells (Fig. 3a). In addition, we found expression of the ectonucleotidase CD73 that, together with CD39, generates extracellular adenosine to inhibit both NK cells and T cells via purinergic receptors 29, as well as expression of CD200 and CD47, modulators of myeloid cell activity (Fig. 3a). Ligands of NK cell activation receptors, such as MICA/B (NKG2D ligand) and CD112 (a DNAM‐1 ligand), as well as CD80 (a T cell co‐stimulator), were detected, together with CD54 [intercellular adhesion molecule (ICAM)‐1] and CD50 (ICAM‐3); ligands of lymphocyte function‐associated antigen 1 (LFA‐1) required for NK cell and T cell‐mediated cytotoxicity 30. The GSC cell surface screen therefore revealed expression of a repertoire of targetable cell surface molecules with the potential to activate and inhibit NK cells, T cells and myeloid cells. This prompted us to explore the expression of immunosuppressive pathways in more detail, using a publicly available GBM single‐cell gene expression data set 18. Among 3589 single cells, we identified 757 co‐expressing SOX2 and EGFR (defined by Darmanis *et al.* 18 as tumour cells) and 1527 cells expressing PTPRC (encoding CD45, a marker of cells of haematopoietic origin.) We next performed unsupervised hierarchial clustering using expression of lineage marker genes and genes encoding candidate immunosuppressive functions, which identified two main groups: non‐immune (comprising tumour and neuronal cells) and immune cells (PTPRC^+^) (Fig. 3b). The immune cell group was dominated by expression of numerous myeloid cell markers (Fig. 3b). Genes encoding immunosuppressive functions were expressed within both the immune and non‐immune clusters (Fig. 3b) and, overall, individual immune cells expressed a greater number of immunosuppressive genes than tumour cells (Supporting information, Fig. S3A). Consistent with the altered cell surface phenotype of GBM‐resident NK cells (Fig. 2a), we found widespread expression of TGFB family transcripts accounted for by TGFB1 expression in the myeloid cells and TGFB2 and TGFB3 expression in non‐immune cells. Furthermore, human leucocyte antigen G (HLA‐G) (which plays a key role in regulating NK cell activity in pregnancy and cancer 31, 32) was also widely expressed. The HLA‐G protein inhibits myeloid cells via receptors leucocyte immunoglobulin‐like receptor subfamily B member 1 (LILRB)1 and LILRB2 31, both of which were expressed in the immune compartment at the mRNA level. We identified strong expression of the receptor‐ligand pair hepatitis A virus cellular receptor 1 (HAVCR1) (TIM3) and lectin, galactose binding, soluble 9 (LGALS9) in the myeloid cluster (92% of immune cells expressed HAVCR2 or LGALS9 and 60% expressed both genes; Supporting information, Fig. S3B). This scRNA‐seq data along with the GSC surface antigen screen shows that both tumour and immune infiltrating cells express receptors and ligands that together constitute a complex network of immunosuppression. For example, the combined action of CD73 and CD39 generate immunosuppressive adenosine 29; our data show expression of CD73 by the tumour cells (Fig. 3a) and 5'‐nucleotidase ecto (NT5E) (encoding CD39) by the immune fraction (Fig. 3b), with Mohme *et al.* demonstrating CD39 expression by GBM‐infiltrating T cells 13.

Furthermore, to assess whether this immunosuppressive network was induced in response to tumour, we analysed gene expression data derived from normal brain tissue 33 (Supporting information, Fig. S4). Microglia/macrophages (the only cell population in the normal brain expressing PTPRC/CD45) constitutively express several immunosuppressive genes, including immune checkpoints V‐set immunoregulatory receptor (VSIR) and HAVCR2 and checkpoint ligands LGALS9, CD274 and PDCD1LG2. Some components of the immunosuppressive network found in brain tumours are therefore present in the healthy brain.

### A spectrum of immune activity in GBM patients

**Figure 4:**
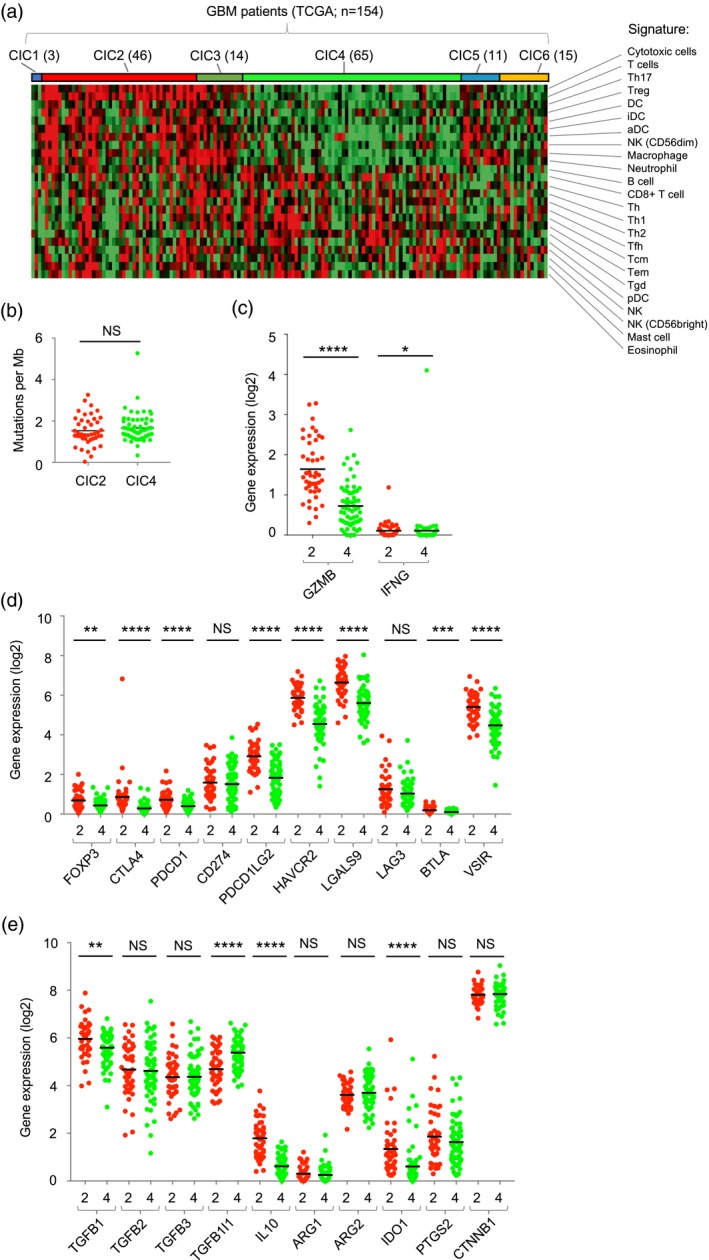
A spectrum of immune activity in glioblastoma (GBM). (a) Classification of GBM tumours [from 154 patients in the The Cancer Genome Atlas (TCGA) data set] into consensus immunome clusters (CIC) using the nearest centroid classification. The number of patients in each CIC is indicated in brackets. The cell signatures used to derive the CIC [14] are shown. (b) Mutational load in GBM CIC2 (red) and CIC4 (green) expressed as mutations per megabase. (c) Expression of granzyme B (GZMB) and interferon gamma (IFN‐γ) in CIC2 (red) and CIC4 (green). (d) Expression of negative regulators of immunity in CIC2 (red) and CIC4 (green). (e) Expression of cytokines and enzymes associated with immunosuppressive activity. (b–e) Data from CIC2 and CIC4 were compared using the Mann–Whitney test; n.s. = not significant, *P < 0·05, **P < 0·01, ***P < 0·001, ****P < 0·0001.

We have previously used tumour transcriptome data to cluster melanoma patients according to their immune cell infiltrate 14. This approach identified six CICs, with one cluster enriched in cytotoxic cells (CIC2) and another (CIC4) having low immune infiltrates and significantly worse survival 14. We used this approach to classify GBM transcriptome data (from TCGA) and, like the situation in melanoma, the cohort of 154 patients clustered into the six CICs (Fig. 4a), with two main clusters CIC2 (high immune infiltrate) and CIC4 (low immune infiltrate). We found that CIC2 was significantly enriched for tumours of the mesenchymal subtype 34 (Supporting information, Table S2) that has been previously shown to have prolonged survival 21. However, unlike melanoma 14, immune infiltration (reflected in the CIC clusters) was not associated with significant differences in survival in GBM (Supporting information, Fig. S5). There was also no significant difference in mutation burden, a surrogate of neoantigen load reflecting immunogenicity 8, 9, between CIC2 and CIC4 in GBM (Fig. 4b). These data demonstrate that patients can be stratified based on the immune infiltrate but that, unlike melanoma, this stratification has no effect on patient outcomes under the conditions of treatment currently employed.

Immune activation induces feedback inhibitory pathways, including the expression of immune checkpoint molecules, and we therefore attempted to use the expression of genes in these pathways to understand the immune environment within the GBM CIC clusters. To do this we compared expression of anti‐tumour effector functions and immunomodulatory genes in CIC2 and CIC4. Expression of the granzyme B (GZMB) and interferon (IFN)‐γ genes were significantly higher in CIC2 than CIC4, consistent with the increased infiltration of cytotoxic T cells and NK cells (Fig. 4c). Furthermore, genes encoding immune checkpoint molecules (CTLA‐4, PDCD1, HAVCR2, BTLA and VSIR), their ligands (PDCD1LG2, LGALS9) and forkhead box protein 3 (FoxP3) were also expressed at significantly higher levels in CIC2 than CIC4 (Fig. 4d), as were genes encoding soluble mediators of immunosuppression such IL‐10, TGF‐β1 and IDO1 (Fig. 4e). To confirm this we used microarray data from the REMBRANDT study 17 and GZMA gene expression as a simple surrogate for immune infiltration 35. This analysis confirmed the significantly higher expression of multiple immunosuppressive functions in patients with increased expression of anti‐tumour effector functions (Supporting information, Fig. S6). Collectively, these data drive our understanding of the GBM immune microenvironment, demonstrating a spectrum of immune infiltration, functionally compromised by an active immune‐inhibitory network.

## Discussion

Our analysis demonstrates tumour and immune‐mediated immunosuppression within the GBM tumour microenvironment, functionally inactivating GBM anti‐tumour immunity. We demonstrate reduced expression of tumour‐sensing activating receptors on GBM‐resident NK cells consistent with TGF‐β activity 26. The TGF‐β family cytokines play a manifold role in glioma progression, including maintenance of the GSC pool, proliferation, invasion, angiogenesis and immunosuppression 36. Multiple mechanisms of tumour‐mediated down‐regulation of NK cell activation receptors have been identified. However, we favour TGF‐β as a modulator of the NK cell phenotype in GBM, as we show reduced expression of activation receptors coupled with increased expression of CD9, a tetraspanin induced by TGF‐β in NK cells 28. Mohme *et al.* showed that infiltrating T cells expressed PD‐1, TIM‐3 and CD39 13, characteristic of T cell exhaustion 37. Our analysis extended these findings by identifying CTLA‐4 and LAG‐3 on GBM‐infiltrating T cells. Thus, GBM‐infiltrating NK cells have reduced expression of activating receptors, whereas T cells have increased expression of immune checkpoint molecules, resulting in inhibition of both classes of cytotoxic lymphocytes. Furthermore, we identified CD73 on the GSC cell surface, and together with CD39 on infiltrating T cells these ectonucleosidases may act together to generate immunosuppressive adenosine which inhibits both NK cells and T cells 29. Similar to Castriconi *et al.* 38, we demonstrate that activated NK cells are capable of recognizing and killing GSC cell lines *in vitro*, and we further show that NK cells discriminate between the GSC and a normal neural progenitor cell line.

The analysis of NK cells in GBM and their interaction with GSCs led to a more extensive analysis of the immunosuppressive network. Our data highlight the abundance of immunosuppressive pathways operating in GBM. The most abundant immune cells in GBM belong to the myeloid lineage 12, and we show that GSCs express cell surface molecules such as PD‐L1 and CD47, which inhibit phagocytosis by macrophage 18, 39, 40. These data demonstrate that immune inhibition within GBM is mediated by both immune and non‐immune lineages co‐operating to provide a pro‐tumour environment. Interestingly, CD47 and CD200 are important regulators of microglial activity and brain inflammation in non‐malignant disease 41. The single‐cell transcriptome data reveal the extent of candidate pathways operating in GBM. Many of these molecules have been detected at the protein level on GBM tumour cells and immune infiltrates, both in this study using flow cytometry of GSCs and infiltrating lymphocytes (e.g. immune checkpoints, checkpoint ligands and ectonucleosidases), as well as in previous studies, using immunohistochemistry and flow cytometry 42, 43, 44, 45, 46.

Several of the immunosuppressive pathways evident in GBM are in place in the normal brain. In response to TGF‐β, microglia suppress immunological activity and promote normal microglial functions such as synaptic pruning and neuronal growth support 47. The expression of genes such as HAVCR2 and its ligand LGALS9 and CD274, together with TGFB2 and IL‐10, safeguard the normal brain against excessive inflammation 48. Muller *et al*. demonstrate that infiltrating macrophages rather than resident microglia encode immunosuppressive cytokines within the GBM microenvironment 49. Thus, immunosuppressive pathways operating in the GBM‐free brain are utilized and extended upon by infiltrating myeloid cells, contributing to the extensive immunosuppressive network.

The identification of a spectrum of immune infiltration across the GBM cohort, accompanied by evidence of anti‐tumour effector function and feedback inhibitory pathways, suggests that GBM should not simply be regarded as an immunogenically ‘cold’ tumour. The high expression of mutiple feedback inhibitors, together with high expression of GZMB and IFN‐γ in CIC2, identifies ongoing, or at least prior, immune activation in a subset of GBM patients, restrained by the action of these inhibitory pathways.

Indeed, melanoma shows interpatient heterogeneity of immune responses, and regarding melanoma as ‘hot’ fails to account for this variability. In melanoma, immune heterogeneity impacts upon survival 14 and the success of immunotherapy, with high expression of PD‐1, CD8^+^ T cell infiltration and higher mutational burden associating with response to therapy 9. We found no evidence for differential survival in GBM according to our CIC classifications, suggesting that the extensive immunosuppressive network removes any impact of immune control on GBM progression. However, these survival data are based on standard therapy and, by analogy with melanoma, we suggest that GBM CIC2 patients are more likely to respond to immunotherapy. Moreover, the extensive immunosuppressive network suggests that targeting multiple inhibitory pathways will be a probable requirement of GBM immunotherapy.

The mechanisms underlying interpatient heterogeneity of immune response in GBM are unclear 50. β‐catenin‐mediated immune evasion pathways operate in CIC4, the group with the poorest prognosis in melanoma 14, whereas in this study we found no evidence of CTNNB1 differential expression between CIC2 and CIC4, nor was their mutational burden significantly different. However, GBM arises through various combinations of oncogene and tumour suppressor mutations, and several of these genes regulate tumour immune responses 51, 52. Thus, differences in oncogene and/or tumour suppressor gene mutations between patients is one potential factor underlying the spectrum of immune activity seen across the GBM cohort.

GSCs are effective targets of NK cells *ex vivo*, but GBM‐infiltrating NK cells have a surface phenotype bearing the hallmarks of TGF‐β‐mediated immunosuppression. Further exploration of immunosuppressive pathways using gene and protein profiling indicated that both tumour and immune cell components contribute inhibitory factors. These pathways are a barrier to effective immunotherapy, but also represent candidate therapeutic targets. Combined checkpoint blockade is already outperforming monotherapy in melanoma 46. Targeting combinations of the multiple immune checkpoints (PD‐1, LAG‐3, CTLA‐4, TIM‐3, VSIR/VISTA) or other immunosuppressive molecules (e.g. ectonucleosidases, TGF‐B, IL‐10) may prove beneficial in GBM. Strategies to activate NK cells *in situ*, e.g. via the use of oncolytic viruses 53, 54, 55 and methods to alter the immune composition of GBM, will also benefit from alleviating key immunosuppressive pathways. Importantly, GBM is not universally devoid of immune activity, and a subset of patients with evidence of immune activity suggests that combinatorial immunotherapy would be most effective when patients are stratified according to immune infiltrates.

## Disclosures

The authors declare that they have no competing interests.

## Author contributions

Experimental design: H. J. C., E. B. W., G. P. C.; implementation: H. J. C., E. B. W. and J. N.; clinical support, including surgery and sample provision: S. C. S., A. A. M., R. K. M. and R. C.; analysis and interpretation of the data: H. J. C., E. B. W., G. P. C., L. F. S., J. N., A. D., H. W., J. N.‐B., S. C. S. and A. A.; GSC provision: H. W.; informatics and statistical analyses: L. F. S., J. N., K. A. R., A. D. and G. P.C.; manuscript preparation: H. J.C., G. P. C. and E. B. W., with input from all authors.

## Supporting information


**Figure S1.** Gating strategy for identification of T cells and NK cells in blood (top) and tumour (bottom) samples

**Figure S2.** The cell surface phenotype of GBM‐infiltrating lymphocytes

**Figure S3.** Expression of immunomodulatory molecules in GBM single cell RNAseq data

**Figure S4.** Gene expression in brain cell populations in the absence of tumour cells.

**Figure S5.** Survival of GBM patients classified according to CIC

**Figure S6.** Microarray data from 214 GBM patients in the REMBRANDT study

**Table S1.** Percentage expression of selected antigen expressed on the surface of human GBM stem‐like cells GBM4, GBM11 and GBM13

**Table S2.** The number of TCGA patient tumours classified into each GBM expression subtype per immune group
