# Supplementary material for: Expression profiling of single cells and patient cohorts identifies multiple immunosuppressive pathways and an altered NK cell phenotype in glioblastoma
Source: Clin Exp Immunol. 2019 Dec 16;200(1):33–44. doi: 10.1111/cei.13403 (PMC7066386; doi:10.1111/cei.13403)
Supplement: Supplementary file 1 — Figure S1. Gating strategy for identification of T cells and NK cells in blood (top) and tumour (bottom) samples [file CEI-200-33-s001.pptx]

## Slide 1
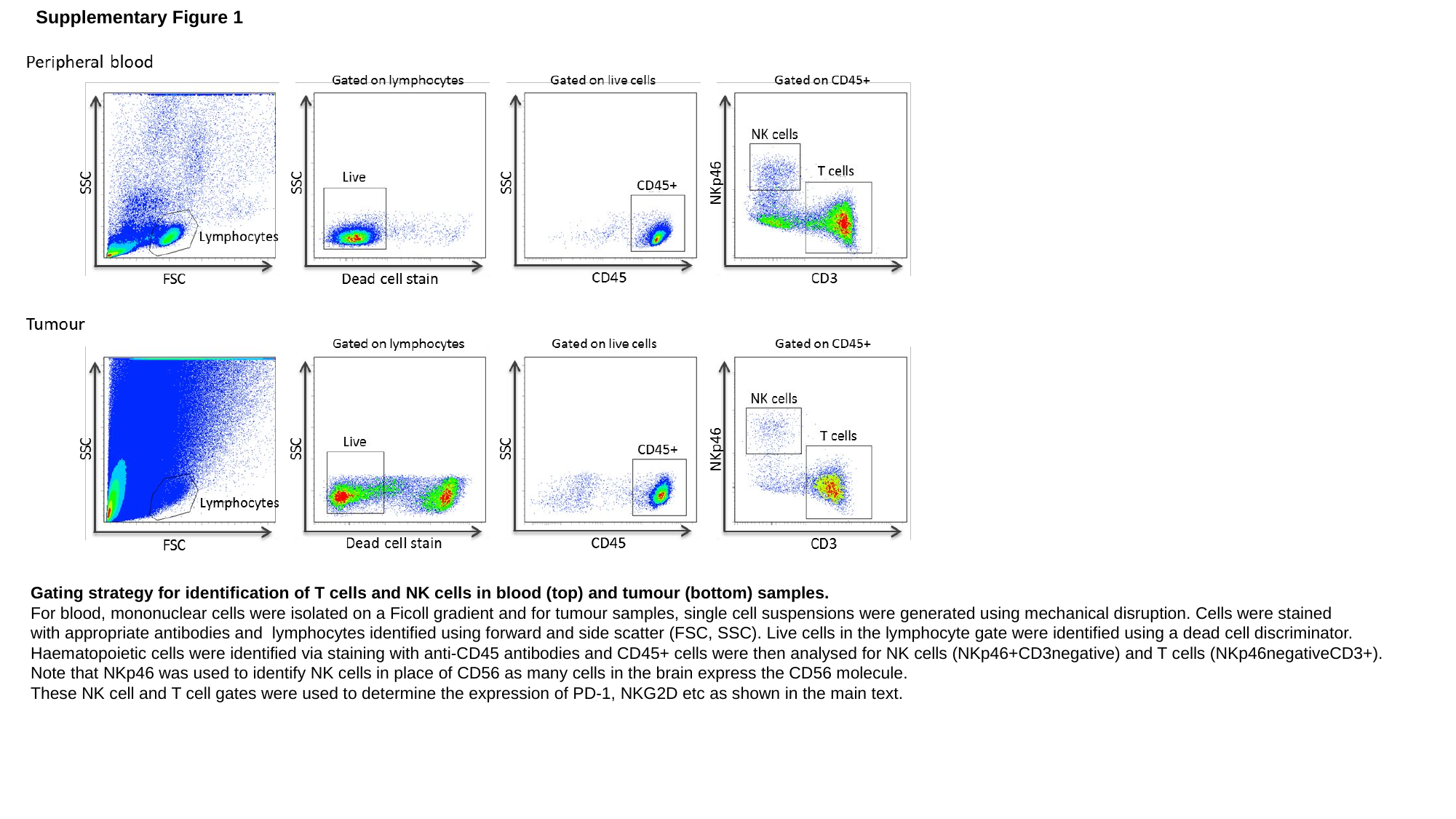

Supplementary Figure 1
Gating strategy for identification of T cells and NK cells in blood (top) and tumour (bottom) samples.
For blood, mononuclear cells were isolated on a Ficoll gradient and for tumour samples, single cell suspensions were generated using mechanical disruption. Cells were stained
with appropriate antibodies and lymphocytes identified using forward and side scatter (FSC, SSC). Live cells in the lymphocyte gate were identified using a dead cell discriminator.
Haematopoietic cells were identified via staining with anti-CD45 antibodies and CD45+ cells were then analysed for NK cells (NKp46+CD3negative) and T cells (NKp46negativeCD3+).
Note that NKp46 was used to identify NK cells in place of CD56 as many cells in the brain express the CD56 molecule.
These NK cell and T cell gates were used to determine the expression of PD-1, NKG2D etc as shown in the main text.
