# Supplementary material for: Expression profiling of single cells and patient cohorts identifies multiple immunosuppressive pathways and an altered NK cell phenotype in glioblastoma
Source: Clin Exp Immunol. 2019 Dec 16;200(1):33–44. doi: 10.1111/cei.13403 (PMC7066386; doi:10.1111/cei.13403)
Supplement: Supplementary file 2 — Figure S2. The cell surface phenotype of GBM‐infiltrating lymphocytes [file CEI-200-33-s002.pptx]

## Slide 1
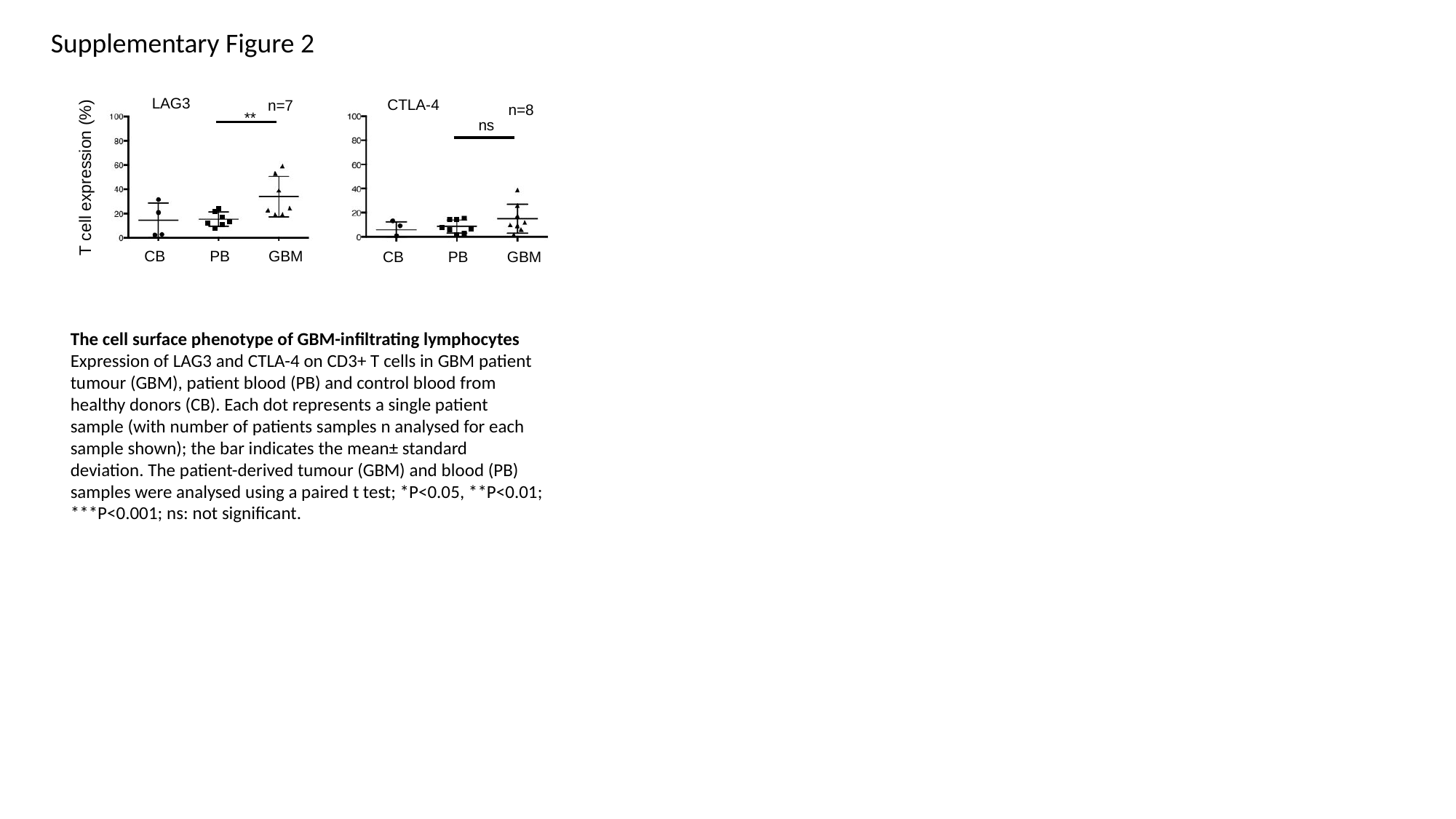

Supplementary Figure 2
LAG3
CTLA-4
**
ns
CB
PB
GBM
CB
PB
GBM
n=7
n=8
T cell expression (%)
The cell surface phenotype of GBM-infiltrating lymphocytes
Expression of LAG3 and CTLA-4 on CD3+ T cells in GBM patient tumour (GBM), patient blood (PB) and control blood from healthy donors (CB). Each dot represents a single patient sample (with number of patients samples n analysed for each sample shown); the bar indicates the mean± standard deviation. The patient-derived tumour (GBM) and blood (PB) samples were analysed using a paired t test; *P<0.05, **P<0.01; ***P<0.001; ns: not significant.
