# Supplementary material for: Expression profiling of single cells and patient cohorts identifies multiple immunosuppressive pathways and an altered NK cell phenotype in glioblastoma
Source: Clin Exp Immunol. 2019 Dec 16;200(1):33–44. doi: 10.1111/cei.13403 (PMC7066386; doi:10.1111/cei.13403)
Supplement: Supplementary file 3 — Figure S3. Expression of immunomodulatory molecules in GBM single cell RNAseq data [file CEI-200-33-s003.pptx]

## Slide 1
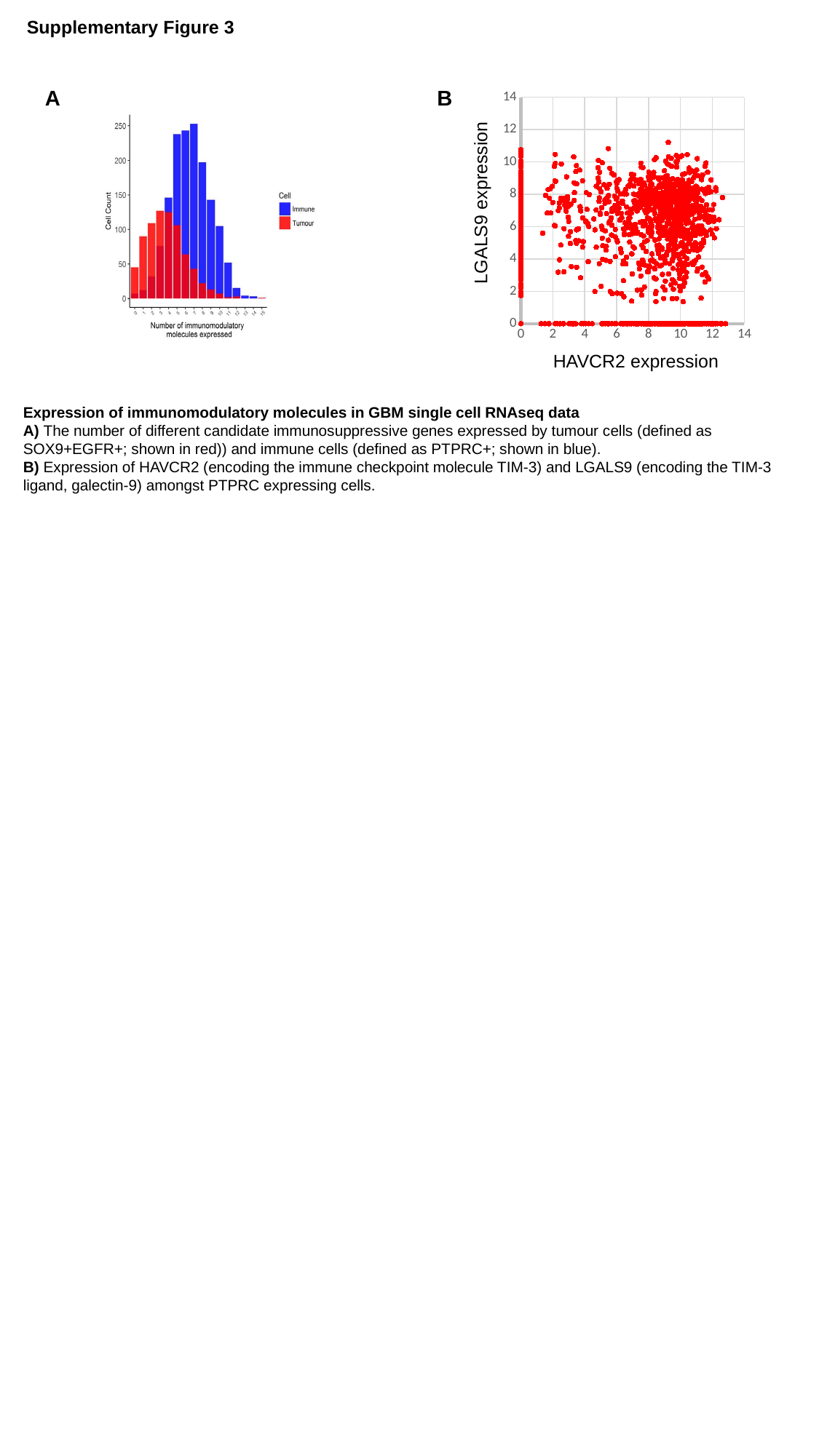

Supplementary Figure 3
A
B
### Chart
| Category | LGALS9 |
|---|---|LGALS9 expression
HAVCR2 expression
Expression of immunomodulatory molecules in GBM single cell RNAseq data
A) The number of different candidate immunosuppressive genes expressed by tumour cells (defined as SOX9+EGFR+; shown in red)) and immune cells (defined as PTPRC+; shown in blue).
B) Expression of HAVCR2 (encoding the immune checkpoint molecule TIM-3) and LGALS9 (encoding the TIM-3 ligand, galectin-9) amongst PTPRC expressing cells.
