# Supplementary material for: Expression profiling of single cells and patient cohorts identifies multiple immunosuppressive pathways and an altered NK cell phenotype in glioblastoma
Source: Clin Exp Immunol. 2019 Dec 16;200(1):33–44. doi: 10.1111/cei.13403 (PMC7066386; doi:10.1111/cei.13403)
Supplement: Supplementary file 4 — Figure S4. Gene expression in brain cell populations in the absence of tumour cells. [file CEI-200-33-s004.pptx]

## Slide 1
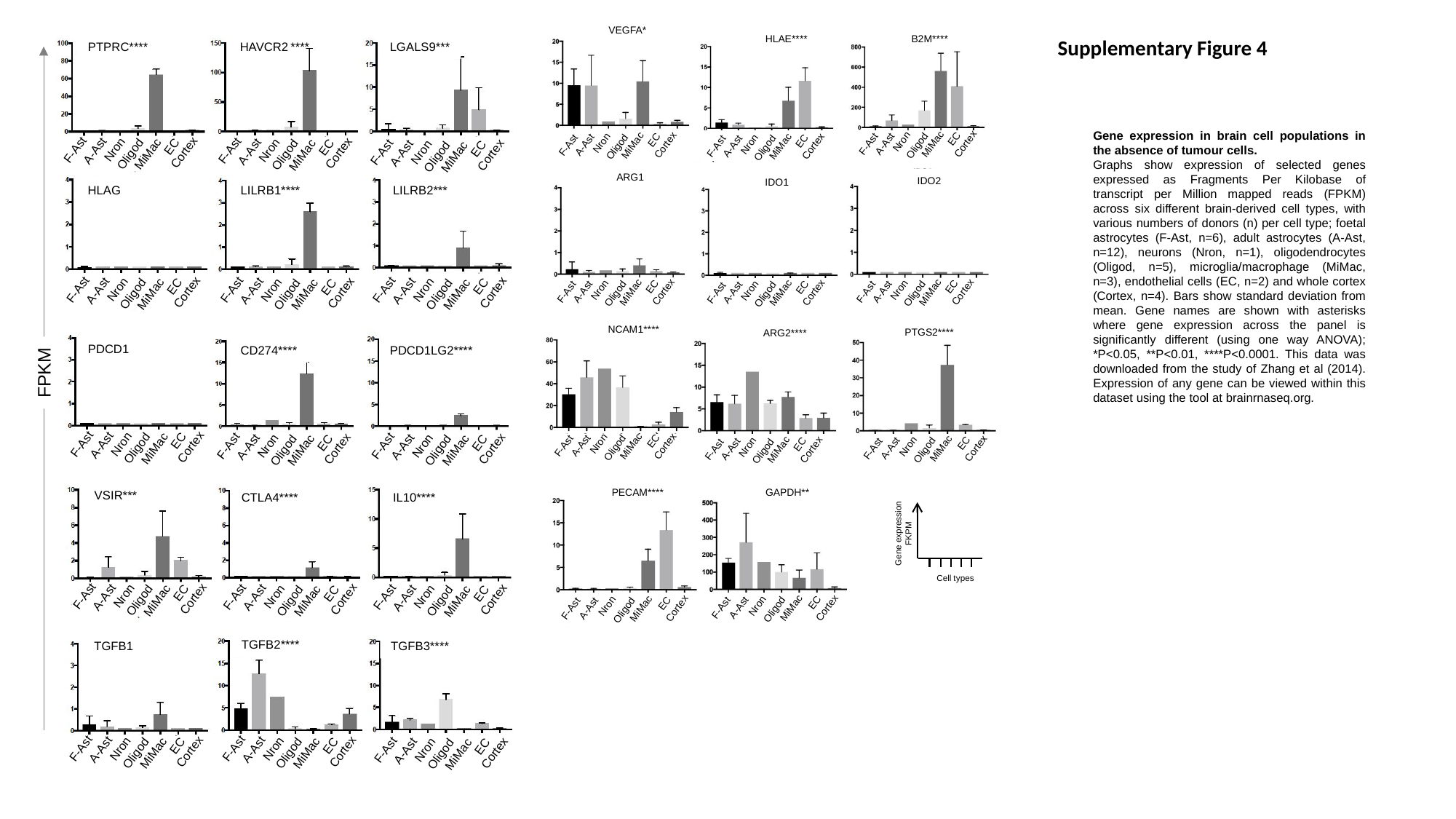

VEGFA*
****
HAVCR2
LGALS9***
PTPRC****
EC
Nron
F-Ast
A-Ast
Cortex
Oligod
MiMac
EC
Nron
F-Ast
A-Ast
Cortex
Oligod
MiMac
EC
Nron
F-Ast
A-Ast
Cortex
Oligod
MiMac
HLAG
LILRB1****
LILRB2***
EC
Nron
F-Ast
A-Ast
Cortex
Oligod
MiMac
EC
Nron
F-Ast
A-Ast
Cortex
Oligod
MiMac
EC
Nron
F-Ast
A-Ast
Cortex
Oligod
MiMac
PDCD1
CD274****
PDCD1LG2****
FPKM
EC
Nron
F-Ast
A-Ast
Cortex
Oligod
MiMac
EC
Nron
F-Ast
A-Ast
Cortex
Oligod
MiMac
EC
Nron
F-Ast
A-Ast
Cortex
Oligod
MiMac
VSIR***
CTLA4****
IL10****
EC
Nron
F-Ast
A-Ast
Cortex
Oligod
MiMac
EC
Nron
F-Ast
A-Ast
Cortex
Oligod
MiMac
EC
Nron
F-Ast
A-Ast
Cortex
Oligod
MiMac
TGFB2****
TGFB1
TGFB3****
EC
Nron
F-Ast
A-Ast
Cortex
Oligod
MiMac
EC
Nron
F-Ast
A-Ast
Cortex
Oligod
MiMac
EC
Nron
F-Ast
A-Ast
Cortex
Oligod
MiMac
HLAE****
B2M****
Supplementary Figure 4
EC
Nron
A-Ast
F-Ast
Cortex
MiMac
Oligod
EC
Nron
A-Ast
F-Ast
Cortex
MiMac
Oligod
EC
Nron
A-Ast
F-Ast
Cortex
MiMac
Oligod
Gene expression in brain cell populations in the absence of tumour cells.
Graphs show expression of selected genes expressed as Fragments Per Kilobase of transcript per Million mapped reads (FPKM) across six different brain-derived cell types, with various numbers of donors (n) per cell type; foetal astrocytes (F-Ast, n=6), adult astrocytes (A-Ast, n=12), neurons (Nron, n=1), oligodendrocytes (Oligod, n=5), microglia/macrophage (MiMac, n=3), endothelial cells (EC, n=2) and whole cortex (Cortex, n=4). Bars show standard deviation from mean. Gene names are shown with asterisks where gene expression across the panel is significantly different (using one way ANOVA); *P<0.05, **P<0.01, ****P<0.0001. This data was downloaded from the study of Zhang et al (2014). Expression of any gene can be viewed within this dataset using the tool at brainrnaseq.org.
ARG1
IDO2
IDO1
EC
Nron
A-Ast
F-Ast
Cortex
MiMac
Oligod
EC
Nron
A-Ast
F-Ast
Cortex
MiMac
Oligod
EC
Nron
A-Ast
F-Ast
Cortex
MiMac
Oligod
NCAM1****
PTGS2****
ARG2****
EC
Nron
A-Ast
F-Ast
Cortex
MiMac
Oligod
EC
Nron
A-Ast
F-Ast
Cortex
MiMac
Oligod
EC
Nron
A-Ast
F-Ast
Cortex
MiMac
Oligod
PECAM****
GAPDH**
Gene expression
FKPM
Cell types
EC
Nron
A-Ast
F-Ast
Cortex
MiMac
Oligod
EC
Nron
A-Ast
F-Ast
Cortex
MiMac
Oligod
