# Supplementary material for: Expression profiling of single cells and patient cohorts identifies multiple immunosuppressive pathways and an altered NK cell phenotype in glioblastoma
Source: Clin Exp Immunol. 2019 Dec 16;200(1):33–44. doi: 10.1111/cei.13403 (PMC7066386; doi:10.1111/cei.13403)
Supplement: Supplementary file 5 — Figure S5. Survival of GBM patients classified according to CIC [file CEI-200-33-s005.pptx]

## Slide 1
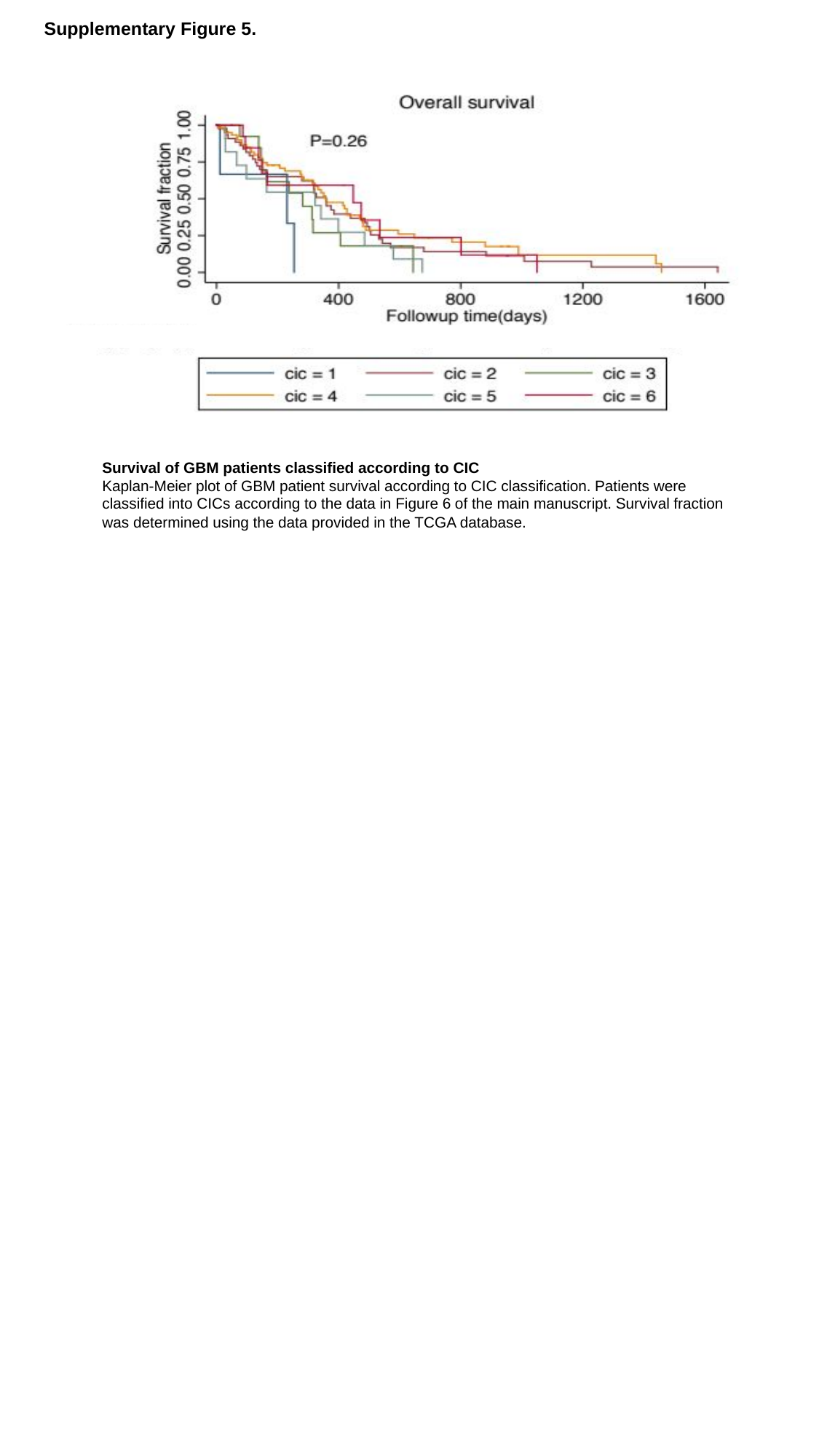

Supplementary Figure 5.
Survival of GBM patients classified according to CIC
Kaplan-Meier plot of GBM patient survival according to CIC classification. Patients were
classified into CICs according to the data in Figure 6 of the main manuscript. Survival fraction
was determined using the data provided in the TCGA database.
