# Supplementary material for: Expression profiling of single cells and patient cohorts identifies multiple immunosuppressive pathways and an altered NK cell phenotype in glioblastoma
Source: Clin Exp Immunol. 2019 Dec 16;200(1):33–44. doi: 10.1111/cei.13403 (PMC7066386; doi:10.1111/cei.13403)
Supplement: Supplementary file 6 — Figure S6. Microarray data from 214 GBM patients in the REMBRANDT study [file CEI-200-33-s006.pptx]

## Slide 1
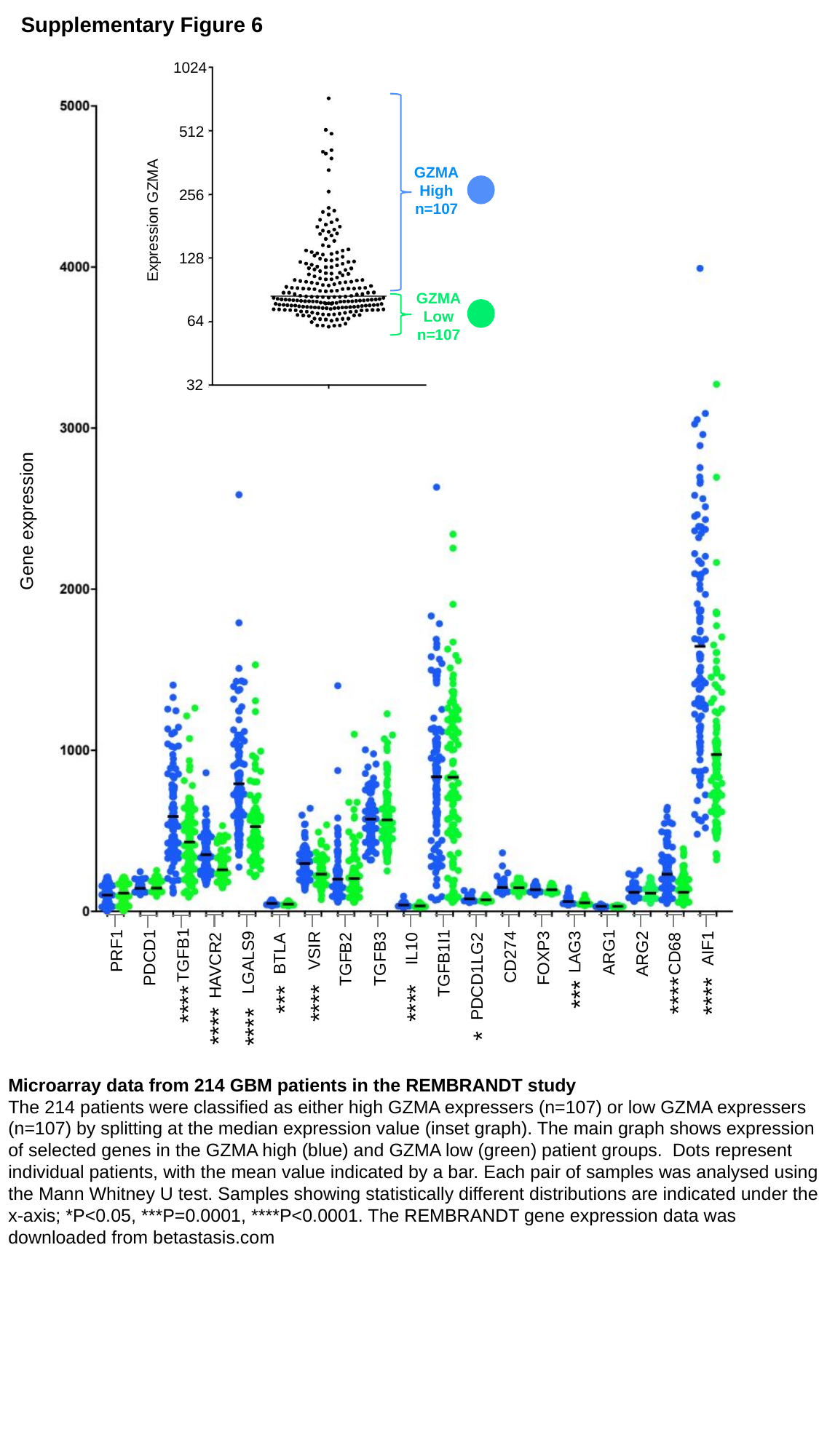

Supplementary Figure 6
1024
512
GZMA
High
n=107
256
Expression GZMA
128
GZMA
Low
n=107
64
32
Gene expression
AIF1
IL10
VSIR
PRF1
LAG3
ARG1
BTLA
CD68
ARG2
TGFB1
CD274
PDCD1
FOXP3
TGFB3
TGFB2
LGALS9
TGFB1I1
HAVCR2
PDCD1LG2
 ***
 ****
 ****
 ***
 ****
 ****
 ****
 ****
 ****
 *
Microarray data from 214 GBM patients in the REMBRANDT study
The 214 patients were classified as either high GZMA expressers (n=107) or low GZMA expressers
(n=107) by splitting at the median expression value (inset graph). The main graph shows expression
of selected genes in the GZMA high (blue) and GZMA low (green) patient groups. Dots represent
individual patients, with the mean value indicated by a bar. Each pair of samples was analysed using
the Mann Whitney U test. Samples showing statistically different distributions are indicated under the
x-axis; *P<0.05, ***P=0.0001, ****P<0.0001. The REMBRANDT gene expression data was
downloaded from betastasis.com
