# Supplementary material for: Expression profiling of single cells and patient cohorts identifies multiple immunosuppressive pathways and an altered NK cell phenotype in glioblastoma
Source: Clin Exp Immunol. 2019 Dec 16;200(1):33–44. doi: 10.1111/cei.13403 (PMC7066386; doi:10.1111/cei.13403)
Supplement: Supplementary file 7 — Table S1. Percentage expression of selected antigen expressed on the surface of human GBM stem‐like cells GBM4, GBM11 and GBM13 [file CEI-200-33-s007.pdf]

Supplementary Table 1

GBM4		GBM11		GBM13	
CD	%Gated	CD	%Gated	CD	%Gated
9	99.96	9	100	9	100
15	28.24	10	48.99	24	78.56
24	99.67	26	70.04	26	70.49
26	45.72	29	39.52	29	91.72
29	94.81	34	99.26	34	55.04
34	54.75	38	36.44	44	100
44	97.35	40	34.05	46	100
46	99.98	44	100	47	99.98
47	100	46	100	50	21.35
54	73.14	47	99.99	54	94.73
55	73.42	54	99.41	55	100
56	69.62	55	98.61	56	99.56
57	98.98	56	99.75	57	96.16
59	99.98	57	88.91	59	99.95
61	31.87	59	100	61	84.77
63	99.92	61	91.58	63	99.92
71	99.75	63	99.96	71	99.55
73	60.17	71	99.99	73	99.97
81	99.8	73	99.98	77	64.76
90	64.35	74	95.29	80	25.44
91	90.62	77	85.22	81	99.83
94	92.85	80	28.97	90	99.06
95	97.53	81	99.84	91	99.25
97	99.99	90	30.33	94	97.98
98	100	91	99.32	95	99.76
99	99.99	94	75.71	97	100
105	98.24	95	98.11	98	100
109	20.36	97	99.98	99	99.99
112	79.71	98	100	104	70.65
118	36.06	99	99.86	105	88.5
119	98.89	104	98.99	108	65.92
130	91.14	105	48.77	112	71.2
141	86	106	98.22	118	44.6
142	92	109	29.73	119	98.85
146	94.54	112	32.23	130	93.38
147	100	119	99.06	141	98.98
151	99.86	130	56.11	142	99.19
152	27.17	141	61.41	144	41.24
164	98.76	142	78.23	146	98.83
165	99.92	146	96.73	147	99.99
166	98.03	147	99.99	151	99.89
184	61.1	151	88.73	152	37.74
200	95.55	164	99.98	164	96.08
209	26.39	165	99.67	165	99.98
220	50.1	166	99.68	166	99.47

221	88.91	184	43.79	184	56.94
227	55.26	201	40.7	200	98.67
268	46.26	205	99.62	209	61.79
271	93.53	221	37.27	220	81.34
274	20.2	227	95.48	221	78.72
338	21.76	268	41.13	227	62.9
340	94.98	271	98.13	268	50.41
107A	73.59	273	81.29	271	99.87
107B	38.39	274	97.28	274	75.63
120A	47.52	321	20.37	321	84.49
140A	50.48	338	38.87	329	34.7
140B	55.76	340	86.48	338	38.32
172B	75.75	107A	99.4	340	97.36
49b	99.99	107B	98.22	107A	88.26
49c	83.81	120A	93.31	107B	54.28
49d	51.39	121B	23.32	120A	71.32
49e	88.34	140A	23.9	140B	37.51
49f	98.52	172B	34.05	172B	89.85
51/61	55.64	49b	99.97	49a	35.63
99R	92.65	49c	100	49b	99.98
b2m	99.81	49d	96.86	49c	100
egfr	31.04	49e	100	49d	23.75
GD2	92.33	49f	99.67	49e	99.93
HLA ABC	99.97	51/61	96.93	49f	99.77
HLA DQ	93.52	62P	20.62	51/61	93.03
HLAA2	97.18	99R	88.77	99R	99.45
MICA/B	40.37	b2m	99.96	b2m	99.94
SSEA1	31.86	GD2	99.94	CD326	63.61
		HLA ABC	99.97	egfr	53.2
		HLA DQ	99.46	GD2	99.96
		HLA DR	99.98	HLA ABC	99.99
		HLA DRPQ	99.95	HLA DQ	99.72
				HLAA2	99.22
				MICA/B	43.86
				SSEA4	32.18

GBM1

CD	%Gated
9	26.7
15	36.8
26	32
29	62
34	3.8
44	71.6
46	89.3
47	91.6
54	23.6
56	82.7
57	71.3
58	77.2
58	77.2
59	80.9
63	83.2
71	87.1
73	37.9
81	89.8
90	81.3
91	57.7
94	70.5
95	73.8
97	86
98	75.6
99	87.2
106	25.6
119	75.1
142	82.6
146	83.3
147	90.9
151	89
164	76.7
165	87.8
166	87.3
200	70.6
227	50.5
271	53.4
321	21.5
340	26.8
107a	41.4
140a	28.2
49a	34.9
49b	89.1
49c	29.2
49d	31
49e	68.6

GBM20

CD	%Gated
9	99.97
15	67.12
24	89.22
26	55.02
29	65.05
34	56.14
44	99.84
46	99.98
47	100
50	31.08
54	38.51
55	34.17
56	99.75
57	99.75
58	100
59	99.92
61	88.07
63	99.95
71	99.64
73	99.87
74	37.5
75	22.84
77	65.45
80	57.11
81	100
88	47.35
90	99.04
91	94.09
94	89.63
95	96.69
97	99.97
98	100
99	99.99
105	74.88
106	45.03
108	75.15
112	55.1
118	20.41
119	98.14
130	98.03
138	47.21
141	94.31
142	97.51
146	96.92
147	100
151	99.98

49f	63.8
99r	36.7
b2m	83.8
gd2	53.8
HLA ABC	90.8
HLA DQ	40.3
ssea1	58.9

152	75.76
164	97.38
165	99.97
166	94.15
171	36.09
184	47.56
200	98.92
205	20.88
209	47.54
220	81.45
221	58
227	91.57
231	21.87
268	73.6
271	98.5
273	34.4
274	72.22
321	42.19
338	90.98
340	79.74
107A	78.66
107B	71.14
120A	69.01
120B	41.99
140A	26.31
140B	66.96
172B	70.02
49b	99.33
49c	99.96
49d	82.31
49e	99.83
49f	93.66
51/61	94.17
99R	97.86
b2m	100
egfr	68.68
GD2	99.82
HLA ABC	99.95
HLA DQ	98.28
HLA DR	96.45
HLA DRPQ	95.51
HLAA2	99.94
MICA/B	77.53
SSEA1	66.06

Full list of antigen screened can be found
http://www.bdbiosciences.com/ds/pm/others/23-10930.pdf
Data shows percentage expressed of each antigen (CD).
