# Supplementary material for: Expression profiling of single cells and patient cohorts identifies multiple immunosuppressive pathways and an altered NK cell phenotype in glioblastoma
Source: Clin Exp Immunol. 2019 Dec 16;200(1):33–44. doi: 10.1111/cei.13403 (PMC7066386; doi:10.1111/cei.13403)
Supplement: Supplementary file 8 — Table S2. The number of TCGA patient tumours classified into each GBM expression subtype per immune group [file CEI-200-33-s008.pptx]

## Slide 1
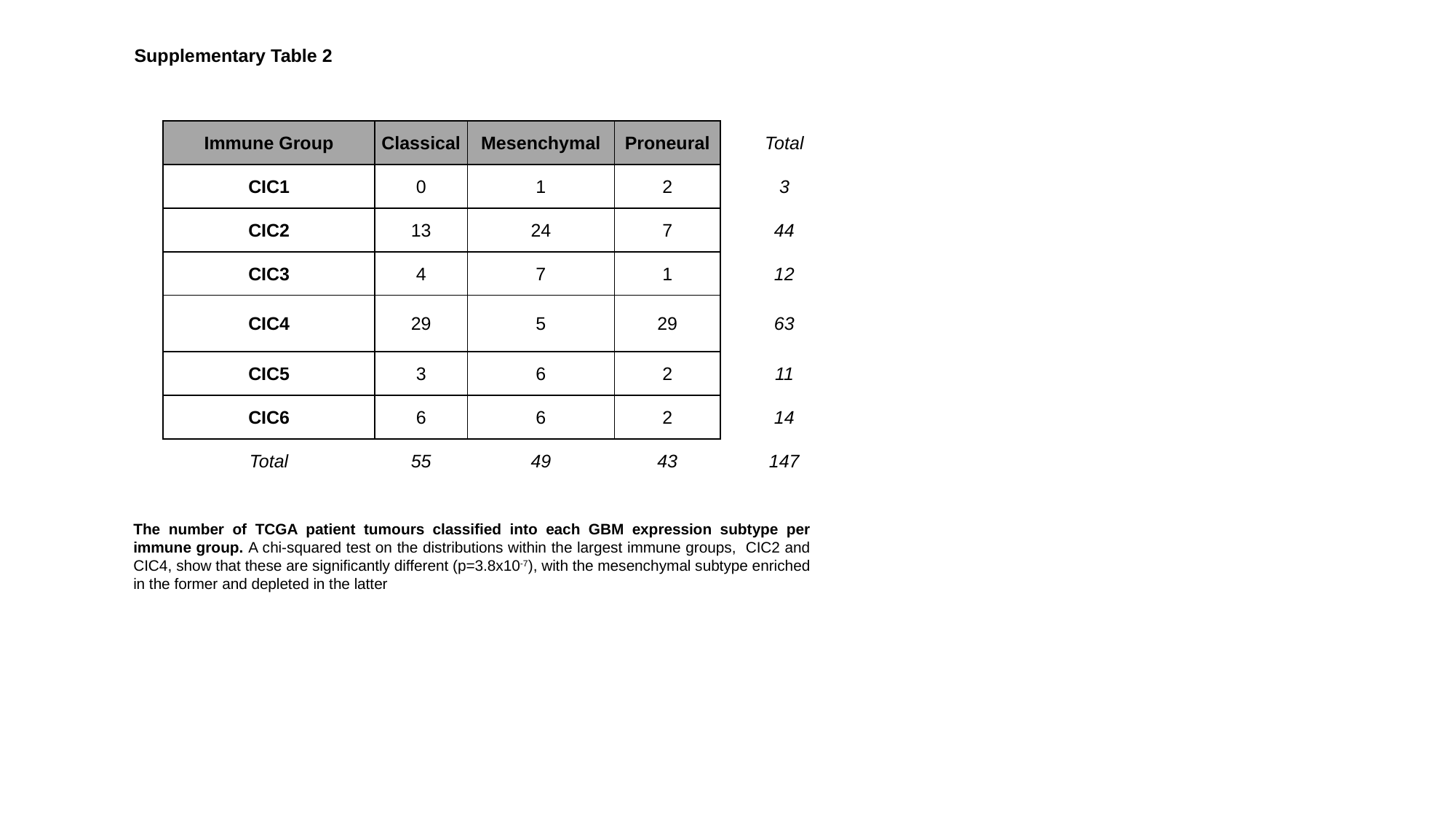

Supplementary Table 2
| Immune Group | Classical | Mesenchymal | Proneural | Total |
| --- | --- | --- | --- | --- |
| CIC1 | 0 | 1 | 2 | 3 |
| CIC2 | 13 | 24 | 7 | 44 |
| CIC3 | 4 | 7 | 1 | 12 |
| CIC4 | 29 | 5 | 29 | 63 |
| CIC5 | 3 | 6 | 2 | 11 |
| CIC6 | 6 | 6 | 2 | 14 |
| Total | 55 | 49 | 43 | 147 |
The number of TCGA patient tumours classified into each GBM expression subtype per immune group. A chi-squared test on the distributions within the largest immune groups, CIC2 and CIC4, show that these are significantly different (p=3.8x10^-7^), with the mesenchymal subtype enriched in the former and depleted in the latter
