# Supplementary material for: Expression profiling of single cells and patient cohorts identifies multiple immunosuppressive pathways and an altered NK cell phenotype in glioblastoma
Source: Clin Exp Immunol. 2019 Dec 16;200(1):33–44. doi: 10.1111/cei.13403 (PMC7066386; doi:10.1111/cei.13403)
Supplement: Supplementary file 9 [file CEI-200-33-s009.pptx]

## Slide 1
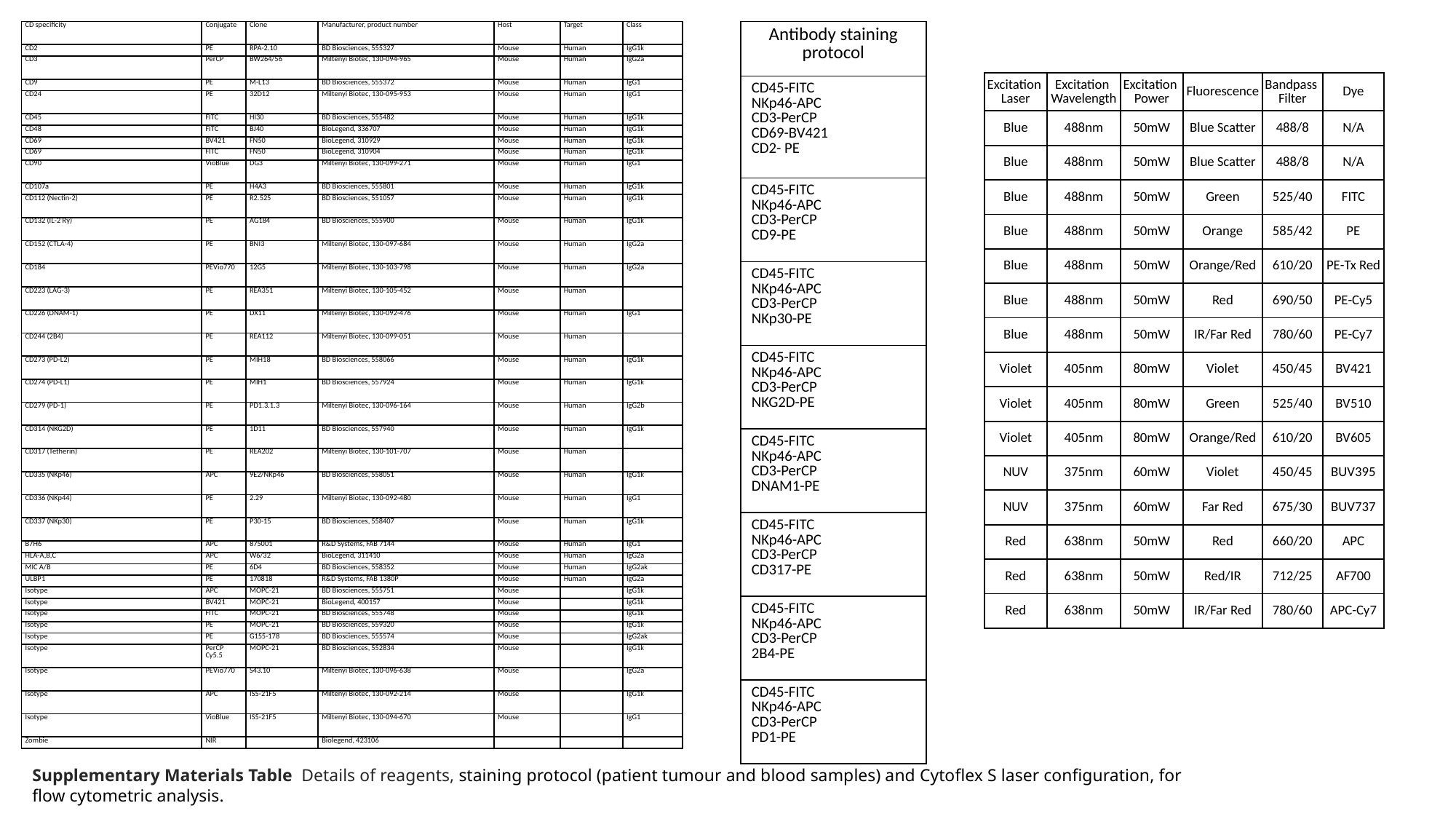

| CD specificity | Conjugate | Clone | Manufacturer, product number | Host | Target | Class |
| --- | --- | --- | --- | --- | --- | --- |
| CD2 | PE | RPA-2.10 | BD Biosciences, 555327 | Mouse | Human | IgG1k |
| CD3 | PerCP | BW264/56 | Miltenyi Biotec, 130-094-965 | Mouse | Human | IgG2a |
| CD9 | PE | M-L13 | BD Biosciences, 555372 | Mouse | Human | IgG1 |
| CD24 | PE | 32D12 | Miltenyi Biotec, 130-095-953 | Mouse | Human | IgG1 |
| CD45 | FITC | HI30 | BD Biosciences, 555482 | Mouse | Human | IgG1k |
| CD48 | FITC | BJ40 | BioLegend, 336707 | Mouse | Human | IgG1k |
| CD69 | BV421 | FN50 | BioLegend, 310929 | Mouse | Human | IgG1k |
| CD69 | FITC | FN50 | BioLegend, 310904 | Mouse | Human | IgG1k |
| CD90 | VioBlue | DG3 | Miltenyi Biotec, 130-099-271 | Mouse | Human | IgG1 |
| CD107a | PE | H4A3 | BD Biosciences, 555801 | Mouse | Human | IgG1k |
| CD112 (Nectin-2) | PE | R2.525 | BD Biosciences, 551057 | Mouse | Human | IgG1k |
| CD132 (IL-2 Rγ) | PE | AG184 | BD Biosciences, 555900 | Mouse | Human | IgG1k |
| CD152 (CTLA-4) | PE | BNI3 | Miltenyi Biotec, 130-097-684 | Mouse | Human | IgG2a |
| CD184 | PEVio770 | 12G5 | Miltenyi Biotec, 130-103-798 | Mouse | Human | IgG2a |
| CD223 (LAG-3) | PE | REA351 | Miltenyi Biotec, 130-105-452 | Mouse | Human | |
| CD226 (DNAM-1) | PE | DX11 | Miltenyi Biotec, 130-092-476 | Mouse | Human | IgG1 |
| CD244 (2B4) | PE | REA112 | Miltenyi Biotec, 130-099-051 | Mouse | Human | |
| CD273 (PD-L2) | PE | MIH18 | BD Biosciences, 558066 | Mouse | Human | IgG1k |
| CD274 (PD-L1) | PE | MIH1 | BD Biosciences, 557924 | Mouse | Human | IgG1k |
| CD279 (PD-1) | PE | PD1.3.1.3 | Miltenyi Biotec, 130-096-164 | Mouse | Human | IgG2b |
| CD314 (NKG2D) | PE | 1D11 | BD Biosciences, 557940 | Mouse | Human | IgG1k |
| CD317 (Tetherin) | PE | REA202 | Miltenyi Biotec, 130-101-707 | Mouse | Human | |
| CD335 (NKp46) | APC | 9E2/NKp46 | BD Biosciences, 558051 | Mouse | Human | IgG1k |
| CD336 (NKp44) | PE | 2.29 | Miltenyi Biotec, 130-092-480 | Mouse | Human | IgG1 |
| CD337 (NKp30) | PE | P30-15 | BD Biosciences, 558407 | Mouse | Human | IgG1k |
| B7H6 | APC | 875001 | R&D Systems, FAB 7144 | Mouse | Human | IgG1 |
| HLA-A,B,C | APC | W6/32 | BioLegend, 311410 | Mouse | Human | IgG2a |
| MIC A/B | PE | 6D4 | BD Biosciences, 558352 | Mouse | Human | IgG2ak |
| ULBP1 | PE | 170818 | R&D Systems, FAB 1380P | Mouse | Human | IgG2a |
| Isotype | APC | MOPC-21 | BD Biosciences, 555751 | Mouse | | IgG1k |
| Isotype | BV421 | MOPC-21 | BioLegend, 400157 | Mouse | | IgG1k |
| Isotype | FITC | MOPC-21 | BD Biosciences, 555748 | Mouse | | IgG1k |
| Isotype | PE | MOPC-21 | BD Biosciences, 559320 | Mouse | | IgG1k |
| Isotype | PE | G155-178 | BD Biosciences, 555574 | Mouse | | IgG2ak |
| Isotype | PerCP Cy5.5 | MOPC-21 | BD Biosciences, 552834 | Mouse | | IgG1k |
| Isotype | PEVio770 | S43.10 | Miltenyi Biotec, 130-096-638 | Mouse | | IgG2a |
| Isotype | APC | IS5-21F5 | Miltenyi Biotec, 130-092-214 | Mouse | | IgG1k |
| Isotype | VioBlue | IS5-21F5 | Miltenyi Biotec, 130-094-670 | Mouse | | IgG1 |
| Zombie | NIR | | Biolegend, 423106 | | | |
| Antibody staining protocol |
| --- |
| CD45-FITC NKp46-APC CD3-PerCP CD69-BV421 CD2- PE |
| CD45-FITC NKp46-APC CD3-PerCP CD9-PE |
| CD45-FITC NKp46-APC CD3-PerCP NKp30-PE |
| CD45-FITC NKp46-APC CD3-PerCP NKG2D-PE |
| CD45-FITC NKp46-APC CD3-PerCP DNAM1-PE |
| CD45-FITC NKp46-APC CD3-PerCP CD317-PE |
| CD45-FITC NKp46-APC CD3-PerCP 2B4-PE |
| CD45-FITC NKp46-APC CD3-PerCP PD1-PE |
| Excitation Laser | Excitation Wavelength | Excitation Power | Fluorescence | Bandpass Filter | Dye |
| --- | --- | --- | --- | --- | --- |
| Blue | 488nm | 50mW | Blue Scatter | 488/8 | N/A |
| Blue | 488nm | 50mW | Blue Scatter | 488/8 | N/A |
| Blue | 488nm | 50mW | Green | 525/40 | FITC |
| Blue | 488nm | 50mW | Orange | 585/42 | PE |
| Blue | 488nm | 50mW | Orange/Red | 610/20 | PE-Tx Red |
| Blue | 488nm | 50mW | Red | 690/50 | PE-Cy5 |
| Blue | 488nm | 50mW | IR/Far Red | 780/60 | PE-Cy7 |
| Violet | 405nm | 80mW | Violet | 450/45 | BV421 |
| Violet | 405nm | 80mW | Green | 525/40 | BV510 |
| Violet | 405nm | 80mW | Orange/Red | 610/20 | BV605 |
| NUV | 375nm | 60mW | Violet | 450/45 | BUV395 |
| NUV | 375nm | 60mW | Far Red | 675/30 | BUV737 |
| Red | 638nm | 50mW | Red | 660/20 | APC |
| Red | 638nm | 50mW | Red/IR | 712/25 | AF700 |
| Red | 638nm | 50mW | IR/Far Red | 780/60 | APC-Cy7 |
Supplementary Materials Table Details of reagents, staining protocol (patient tumour and blood samples) and Cytoflex S laser configuration, for flow cytometric analysis.
